# Two novel C-terminal frameshift mutations in the β-globin gene lead to rapid mRNA decay

**DOI:** 10.1186/s12881-017-0428-1

**Published:** 2017-06-08

**Authors:** Katarzyna Rawa, Roman J. Szczesny, Ewelina P. Owczarek, Anna Adamowicz-Salach, Anna Klukowska, Urszula Demkow, Danuta Plochocka, Pawel Szczesny, Monika Gora, Andrzej Dziembowski, Beata Burzynska

**Affiliations:** 1 Institute of Biochemistry and Biophysics, Polish Academy of Sciences, Pawinskiego 5a, 02-106 Warszawa, Poland; 2 Institute of Genetics and Biotechnology, Faculty of Biology, University of Warsaw, 02-106 Warsaw, Poland; 3 Department of Paediatrics, Haematology and Oncology, Medical University of Warsaw, 02-091 Warsaw, Poland; 4 Department of Laboratory Diagnostics and Clinical Immunology of Developmental Age, Medical University of Warsaw, 02-091 Warsaw, Poland

**Keywords:** β- thalassemia, Frameshift mutations, Gene expression, mRNA degradation

## Abstract

**Background:**

The thalassemia syndromes are classified according to the globin chain or chains whose production is affected. β-thalassemias are caused by point mutations or, more rarely, deletions or insertions of a few nucleotides in the β-globin gene or its immediate flanking sequences. These mutations interfere with the gene function either at the transcriptional, translational or posttranslational level.

**Methods:**

Two cases of Polish patients with hereditary hemolytic anemia suspected of thalassemia were studied. DNA sequencing and mRNA quantification were performed. Stable human cell lines which express wild-type *HBB* and mutated versions were used to verify that detected mutation are responsible for mRNA degradation.

**Results:**

We identified two different frameshift mutations positioned in the third exon of *HBB*. Both patients harboring these mutations present the clinical phenotype of thalassemia intermedia and showed dominant pattern of inheritance. In both cases the mutations do not generate premature stop codon. Instead, slightly longer protein with unnatural C-terminus could be produced. Interestingly, although detected mutations are not expected to induce NMD, the mutant version of mRNA is not detectable. Restoring of the open reading frame brought back the RNA to that of the wild-type level.

**Conclusion:**

Our results show that a lack of natural stop codon due to the frameshift in exon 3 of β-globin gene causes rapid degradation of its mRNA and indicate existence of novel surveillance pathway.

**Electronic supplementary material:**

The online version of this article (doi:10.1186/s12881-017-0428-1) contains supplementary material, which is available to authorized users.

## Background

β-thalassemias are one of the most common monogenic diseases in the world. They are common in tropical and subtropical regions where they provide protection against malaria, but are rather rare in Northern Europe. Some limited cases have been described in the indigenous British, Irish, Breton [1, 2], Scandinavian [3], German [4] and Polish populations [5]. The total annual prevalence of symptomatic individuals is estimated at 1 in 10,000 in the European Union [6].

Hemoglobin disorders comprise two different groups, structural hemoglobin defects and the thalassemias, where the abundance of a globin chain is decreased. The classical recessive forms of β-thalassemia lead to a reduced production of normal β-globin chains. In contrast, dominantly inherited β-thalassemia is caused by some rare mutations resulting in the synthesis of extremely unstable β-globin [7].

The *HBB* gene encoding β-globin, spans 1.6 kb and comprises three exons, with the first and third one containing mainly noncoding sequences of the5’ and 3’ UTRs. 378 different β-thalassemia mutations have now been characterized worldwide [8]. In many cases β-thalassemia mutations generate premature stop codons which lead to degradation of mutated RNA by RNA surveillance mechanism called nonsense-mediated mRNA decay (NMD). Activation of NMD for the β-globin mRNA depends on the position of the nonsense mutations: mutations that reside at least 50 nt5’ to the last intron/exon junction direct the affected mRNA for rapid decay, while those occurring in the 3’part of the gene usually escape NMD [9]. In very rare cases disappearance of mRNA for β-globin in which mutations are present at the 3’ end of the mature mRNA has been described [10]. RNA surveillance mechanism responsible for this phenomenon remains to be determined.

The aim of the present study was to look for mutations in the β-globin gene in patients with hereditary hemolytic anemia suspected of thalassemia. Two cases (Patient 1 and Patient 2) were investigated and various frameshift mutations (c.375_376insCCAGT and c.349del) located in the third exon have been identified. In both patients the level of β-globin mRNA was substantially lower than in healthy subjects. By mimicking CCAGT insertion in cultured human cells we established the molecular mechanism by which the identified genetic changes diminish β-globin expression. Here, we show that newly identified mutations direct the *HBB* mRNA to degradation, which depends on proper position of termination codon.

## Methods

### Subjects

**Patient 1:** A 6-year-old boy, born to non-consanguineous parents has been under medical supervision since birth because of hemolytic anemia of unknown etiology. He presented with splenomegaly and xanthoderma. Occasionally, the patient needed red blood cells (RBCs) transfusions. Laboratory tests indicated profound anemia and hyperbilirubinemia. Congenital dyserythropoietic anemias, hereditary spherocytosis and anemias due to deficiency of red cell enzymes were excluded. HbF and HbA_2_ levels were elevated and β-thalassemia was diagnosed. Compatible clinical presentation of hemolytic anemia was observed in mother and younger sister of the proband. His mother had splenectomy in childhood without significant improvement of anemia but she hasn’t need RBC transfusion. The results of blood tests of all subjects are presented in Table 1.

**Table 1:** Hematologic parameters and molecular data

	Family 1			Family 2	
	Patient 1	Sister of Patient 1	Mother of Patient 1	Patient 2	Brother of Patient 2
Age (years)	6	1.2	38	10	2
RBC (10^6/uL)	2.7	3.4	2.6	2.9	4.7
Hb (g/dL)	6.2	8.7	7.7	7.4	12.4
MCV (fL)	74	80.7	93	90.4	79.4
RDW (%)	34.4	21.3	26.9	35.8	14.3
Retics (% of RBC)	22.60	N.D.	15.83	11.43	1.92
HbA_2_ (%)	3.2	2.2	4.05	4.3	3.4
HbF (%)	9.5	22.5	12.9	4.0	2.1
XmnI polymorphism	+/−	+/−	+/+	−/−	N.D.
β-globin mutation	c.375_376 insCCAGT/β^N^	c.375_376 insCCAGT/β^N^	c.375_376 insCCAGT/β^N^	c.349 delC/β^N^	β^N^/ β^N^

**Patient 2**: A 10-year-old boy with hemolytic anemia and hyperbilirubinemia of unknown etiology, born to healthy non-consanguineous parents, has been observed since birth. Initially, hereditary spherocytosis was diagnosed, based on the assessment of peripheral blood smear. Occasionally, he needed RBC transfusions. At the age of 8, splenectomy was performed but without beneficial effect. Hemoglobin electrophoresis revealed elevated level of fetal hemoglobin (4%) and hemoglobin A_2_ (4.3%), consistently with β-thalassemia diagnosis. There was no family history of blood diseases.

Both patients were Caucasians of Polish origin. The patients' parents gave informed consent to the study. The study protocol has been approved by the Ethical Committee of the Medical University of Warsaw. The study was conducted according to principles laid out in the Declaration of Helsinki.

### RNA isolation, cDNA synthesis and determination of mRNA levels

Total RNA was isolated from peripheral blood samples using the PAXgene Blood RNA Kit (QIAGEN, Hilden, Germany). cDNA was amplified with the use of primers encompassing the coding sequence from exon 2 to 3’UTR. qPCR amplification was performed using a LightCycler1.5 and LightCyclerFastStart DNA Master SYBR Green I (Roche Diagnostics GmbH, Germany).

mRNA quantification of the β-globin genes was carried out using real-time PCR. The Pfaffl model [11] and the relative expression software tool (REST-384©)were used to estimate the relative mRNA level of the β-globin gene in the analyzed patients (compared with 20 healthy individuals). Data normalization was carried out against the transcript of the gene for *EMP55* (erythrocyte membrane protein p55).

### DNA isolation and amplification

Genomic DNA was isolated from peripheral blood using the MagNA Pure Compact Nucleic Acid Isolation Kit I (Roche Diagnostics GmbH, Germany). Polymerase chain reaction (PCR) was used to amplify the promoter region, entire coding sequence and surrounding sequences of the β–globin gene. DNA fragments generated by PCR amplification were sequenced directly as previously described [5]. Primer sequences are given in Additional file 1: Table S1.

### Analysis of XmnI polymorphism

XmnI (Fermentas, Vilnius, Lithuania) was used to verify the presence of the XmnI-Gγ polymorphism [12].

### DNA cloning and site-directed mutagenesis

DNA cloning was performed using standard methods. *HBB* was amplified using RSZ394 (gtcggtaccccatggtgcatctgactcctg) and RSZ396 (gacctcgagttccctttttagtaaaatattcag) primers and DNA isolated from human 293 cells as a template. Amplified DNA fragment was cloned into pcDNA5FRT/TO vector (Invitrogen Carlsbad, CA, USA) digested with Acc65I and XhoI to give plasmid pRS478. Standard site-directed mutagenesis of pRS478 was performed to introduce the mutation identified in patient 1 (pPO9) or the mutation together with an additional nucleotide restoring the reading frame (pPO10). The following primers were used for mutagenesis: RSZ533 (gcaaagaattcaccccaccagtccagtgcaggctgcctatcag) and RSZ534 (ctgataggcagcctgcactggactggtggggtgaattctttgc) (to get pPO9) and RSZ535 (gcaaagaattcaccccaccagtccagttgcaggctgcctatcag) and RSZ536 (ctgataggcagcctgcaactggactggtggggtgaattctttgc) (to get pPO10).

### Cell culture and establishing of stable cell lines

Cells were cultured in DMEM medium (Invitrogen) supplemented with 10% FBS (Invitrogen) under standard conditions (37 °C, 5% CO_2_). Stable transfected cell lines were obtained from human 293 TRex-FlpIn cells using a procedure described previously [13]. Expression of transgenes was induced with tetracycline (25 ng/ml).

### RNA isolation and northern blotting

Total RNA was isolated with TriReagent (Sigma, Steinheim, Germany) according to manufacturer’s recommendation. Northern blotting was performed as previously [13]. The primers used to prepare the probe were RSZ413 (gcaggctgctggtggtcta) and OPE16 (agccaggccatcactaaag).

## Results

Initially we have quantified levels of globin mRNAs in both patients which revealed a decreased β-globin transcript level by more than 50% in the investigated patients and elevated levels of γ- and δ-globin compared to the control group (Table 2).

**Table 2:** Relative mRNA levels of globin genes

	α-globin	β-globin	γ-globin	δ-globin
Patient 1	0.94 (0.001)	0.46 (0.002)	90.89 (0.001)	4.28 (0.003)
Patient 2	1.42 (0.444)	0.37 (0.130)	44.25 (0.003)	44.29 (0.001)

*p*-values are given in brackets

In Patient 2 both γ - and δ -globin mRNAs were substantially more abundant than in controls, while in Patient 1 only γ -globin mRNA was up-regulated markedly and δ-globin one showed a modest increase (Table 2). Additionally, members of the Patient 1 family presented greatly elevated HbF levels (Table 1). One of the most significant genetic factors associated with high HbF is XmnI polymorphism at position −158 upstream of the Gγ-globin gene [14]. We therefore analysed the XmnI polymorphism (Table 1), finding the −158 (C → T) transition in one allele in the Patient 1 patient and his sister. Their mother was homozygous for the XmnI polymorphism (T/T). Patient 2 was homozygous for the −158 C variant, in accordance with his only moderate increased HbF level.

**Fig. 1:**
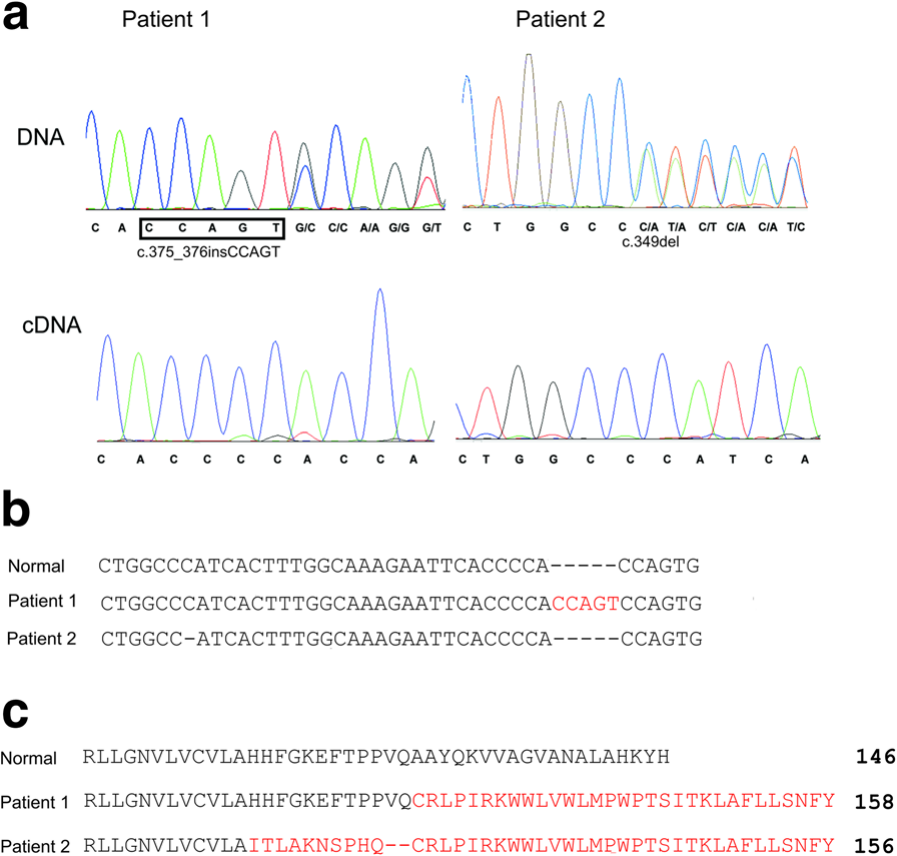
Novel exon 3 mutations in β-globin gene (**a**) DNA and cDNA sequences analysis of Patients 1 and 2 (**b**) Alignment of DNA sequences of normal and mutated β-globin genes from codon 114 to codon 126. (**c**) Amino acid sequences of C-termini of normal β-globin (wt) and hypothetical β-chains starting from last codon of exon 2

In order to identify disease-causing mutations of analyzed patients, their β-globin genes were specifically amplified by PCR and subsequently sequenced. In both patients we found heterozygous mutations in the exon 3 (Fig. 1a, b): in Patient 1 an insertion of five nucleotides (c.375_376insCCAGT) and in Patient 2 a single nucleotide deletion (c.349del). Both these frameshift mutations change position of the stop codon. As a result slightly longer open reading frames are produced. The families of the probands were also tested for the presence of those mutations. The c.375_376insCCAGT mutation was found in the Patient’s 1 sister and mother. For Patient 2, only his brother was available for the study and showed no mutations in the *HBB* gene. The presence of mutations in the investigated DNA samples was confirmed by independent sequencing of the second strand.

To determine whether the mutated alleles are transcribed, we reverse-transcribed region of interest and sequenced the obtained cDNAs. Only the non-mutated sequences were obtained for the both patients, indicating the absence of mRNA encoded by the mutated alleles. This could in principle result from rapid degradation of the aberrant transcript or from lack of transcription of the alleles. The latter seems unlikely, since no addition mutations in the 5’ flank or 5’ part of the gene were detected. Two mutations of the β-globin gene reported here reside in the 5’ part of exon 3 and they both remove the native stop codon at position 147 and create a novel one at positions 159 (Patient 1) or 157 (Patient 2) (Fig. 1c).

**Fig. 2:**
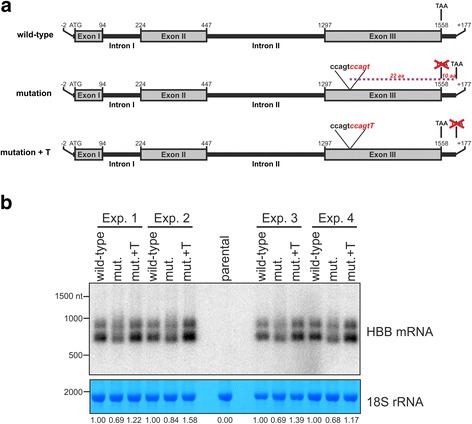
Restoring of original open reading frame rescues of transgene-encoded *HBB* mRNA. **a** Schema of *HBB* transgenes (**b**) Northern blot analysis of *HBB* mRNA encoded by transgenes. RNA (2 μg per sample) isolated from parental 293 cells and their stable transgenic derivatives was resolved by size using gel electrophoresis, transferred to membrane and *HBB* transcripts were detected using radiolabeled probe complementary to exon 2. Lower panels shows RNA methylene blue staining as a loading control. RNA from 4 independent sets of experiments was analyzed. Numbers at the bottom show relative level of the shorter *HBB* mRNA isoform. Signal from 18S rRNA was used for normalization. For a given experiment wild-type sample was regarded as 1

We considered three possible mechanisms which could be responsible for the observed low steady-state levels of the mutated *HBB* mRNA. The mutations could somehow affect transcription of the gene, splicing or the mutated *HBB* mRNA could be degraded rapidly by RNA surveillance. Because, mutations are within a coding region we found the first possibility to be very unlikely. In order to analyse the effect of mutations on splicing and stability we focused on the Patient 1 case (c.375_376insCCAGT) and constructed three stable human cell lines expressing in an inducible manner wild-type *HBB*, its mutated version, or a variant containing the original Patient 1 mutation followed by an additional thymidine which restored the reading frame (Fig. 2a). An incorrect position of the termination codon may lead to RNA surveillance response [13], thus restoring the original reading frame of *HBB* mRNA should rescue the transcript stability. On the other hand if Patient 1 mutation affects processing the restoration of the original open reading frame should not rescue the phenotype. Expression of the transgenes was induced with tetracycline and the levels of corresponding RNAs were analyzed by northern blotting. In agreement with the results for the patient’s blood samples we found that the c.375_376insCCAGT insertion decreases the level of *HBB* RNA in comparison to the wild-type *HBB* (Fig. 2b), however, no effect on splicing was observed. Importantly, the restoring of the original reading frame with the additional nucleotide brought back the RNA to the level to that observed for the wild-type gene (Fig. 2b). The same results were observed for four independent sets of samples. For all the analyzed versions of *HBB* we observed two RNA species of different lengths. Importantly, the level of both species followed the same pattern of changes. These two species could result from alternative transcription termination and/or could reflect different of poly(A) tails. Regardless of the nature of the two *HBB* mRNA species, these results clearly show that frameshift caused by the c.375_376insCCAGT insertion, but not the insertion itself, activates an RNA degradation without affecting pre-mRNA processing.

## Discussion

RNA degradation plays an essential role in ensuring that only fully functional RNA molecules become translated by disposing of aberrant ones that are often mature but dysfunctional. Their expression at the protein level could disturb cellular functioning. Truncated or too long polypeptide chains could titer out other proteins, form inactive complexes, misfold and aggregate or otherwise hamper to the cell. Although numerous mechanisms do exist for the disposal of aberrant polypeptides, they could easily become overwhelmed by excessive production of their substrates. Thus, defective RNA molecules, which are often mature but dysfunctional, need to be degraded rapidly by variety of RNA surveillance pathways [15].

Some cases of β-thalassemias are caused by mutations in the *HBB* gene which introducing a premature stop codon inducing nonsense mediated RNA decay. It has been reported that NMD-promoting mutations located in exon 1 or 2 of β-globin gene subject the transcripts to rapid degradation [16]. Such mutations are associated with mild anemia or even are symptomless. In contrast, mutations in the terminal exon 3 escape NMD and therefore can have a dominant-negative effect [17]. In general, mutations that result in the production of elongated or truncated globin chain manifest in the heterozygous state with a thalassemia intermedia phenotype, frequently associated with the accumulation of inclusion bodies in peripheral erythrocytes. They occur sporadically and show a dominant transmission pattern. In most cases, protein analysis fails to detect the abnormal Hb, presumably owing to its high instability [1].

In our study we identified two different frameshift mutations positioned in the third exon of *HBB*. Both patients harboring these mutations present the clinical phenotype of thalassemia intermedia and showed dominant pattern of inheritance. In both cases the mutations do not generate premature stop codon. Instead, slightly longer protein with unnatural C-terminus could be produced. Interestingly, although mutations of Patient 1 and Patient 2 are not expected to induce NMD, the mutant version of mRNA is not detectable. For one of these mutations, experiments using reporters in 293 cell lines confirmed the degradation of the mutated transcript. Furthermore, restoring of the origin reading frame stabilized the transcript, which suggested that the destruction of the wild type *HBB* reading frame was crucial for the degradation of the defective mRNAs.

Our understanding of the mechanisms controlling RNA quality is limited [18], and the degradation of the mutated β-globin transcripts reported here cannot be explained by any of the current NMD models. Few reports have presented well documented mRNA depletion in thalassemic hemoglobinopathies due to mutations in the third exon. Grosso et al. [10] described a single- nucleotide mutation in exon 3 of the β-globin gene responsible for decreased mRNA levels and a β-thalassemic defect. Musollimo et al. [19] reported that codon 105 + AGCT mutation reduced the level of the abnormal mRNA, which was found present only in trace amounts and could not be translated into a functional β-chain. According to the Globin Gene Server (http://globin.cse.psu.edu/) the majority of frameshift mutations in the exon 3 at codons 108–141 do not produce a Hb variant detectable by electrophoresis, chromatography, or stability tests. In contrast, missense mutations downstream of codon 141 produce abnormal Hb. It seems likely that additional factors could bind to specific sequences created by the frameshift mutations to facilitate their identification and consequent mRNA degradation. It seems that effect of a given mutation, besides other factors, also depends on its position within the *HBB* gene. This phenomenon is currently unclear.

## Conclusion

The two mutations described have add new information regarding degradation of mutated β-globin mRNA and thus could facilitate the deciphering of the underlying mechanism. In particular, we have shown that moving the STOP codon by as few as twelve codons downstream suffices to destabilize the *HBB* mRNA.

## Additional file

Additional file 1: Table S1. Sequences of the primers. (DOCX 13 kb)

## Abbreviations

cDNA: Complementary DNA
HBA_2_: Hemoglobin A2
HBB: Hemoglobin Subunit Beta
HbF: Fetal hemoglobin
mRNA: Messenger RNA
NMD: Nonsense-mediated mRNA decay
PCR: Polymerase Chain Reaction
RBC: Red blood cell
RNA: Ribonucleic acid
UTRs: mRNA Untranslated Regions
