# Supplementary material for: Two novel C-terminal frameshift mutations in the β-globin gene lead to rapid mRNA decay
Source: BMC Med Genet. 2017 Jun 8;18:65. doi: 10.1186/s12881-017-0428-1 (PMC5465470; doi:10.1186/s12881-017-0428-1)
Supplement: Additional file 1: Table S1. — Sequences of the primers. (DOCX 13 kb) [file 12881_2017_428_MOESM1_ESM.docx]

Table S1 Sequences of the primers

1. **Primers used for DNA and cDNA amplification**

| Amplified fragment | Template | Fragment size (bp) | Annealing temperature | Primer sequence 5′-3′ |
| --- | --- | --- | --- | --- |
| 5’UTR,  exon 1-2 | DNA | 763 | 58 | 5’ GTACGGCTGTCATCACTTAG 3’  5’ CCTGAGACTTCCACACTGAT 3’ |
| exon 3,  3’UTR | DNA | 892 | 56 | 5’ CTCTTTCTTTCAGGGCAATA 3’  5’ AGTGGAGTCAAGGCTGAGAG 3’ |
| c.149-c.549 | cDNA | 400 | 56 | 5’ CCACTCCTGATGCTGTTATG 3’  5’ GCAGAATCCAGATGCTCAAG 3’ |

1. **Primers used in qPCR reaction**

| Gene | Primer | Primer sequence 5′-3′ | Fragment size (bp) |
| --- | --- | --- | --- |
| α-globin | globA-F  globA-R | 5’ TGGCCGACGCCCTGACCAA 3’  5’ TCAGCACGGTGCTCACAGA 3’ | 225 |
| β-globin | globB-F  globB-R | 5’ CAAGGGCACCTTTGCCACA 3’  5’ GGCAGAATCCAGATGCTCAAG 3’ | 305 |
| γ-globin | globG-F  globG-R | 5’GAGGACAAGGCTACTATCACAA 3’  5’GGCATCTCCCAAGGAAGTC 3’ | 207 |
| δ-globin | globD-F  globD-R | 5’ CAAGGGCACTTTTTCTCAG 3’  5’ AACAGTCCAGGATCTCAATG 3’ | 213 |
| EMP55 | p55-F  p55-R | 5’ CGGAGGAGATGACGAGGAACA 3’  5’ CCTGGTCAGTAGGTGCAATGAAC 3’ | 210 |
